# Supplementary material for: Operando visualisation of battery chemistry in a sodium-ion battery by 23Na magnetic resonance imaging
Source: Nat Commun. 2020 Apr 29;11:2083. doi: 10.1038/s41467-020-15938-x (PMC7190614; doi:10.1038/s41467-020-15938-x)
Supplement: Supplementary file 1 — Supplementary Information [file 41467_2020_15938_MOESM1_ESM.pdf]

Supplementary Information for

Operando visualisation of battery chemistry in a sodium ion battery by ^{23}Na Magnetic Resonance Imaging

Bray, J. M. et al.

Supplementary Figures

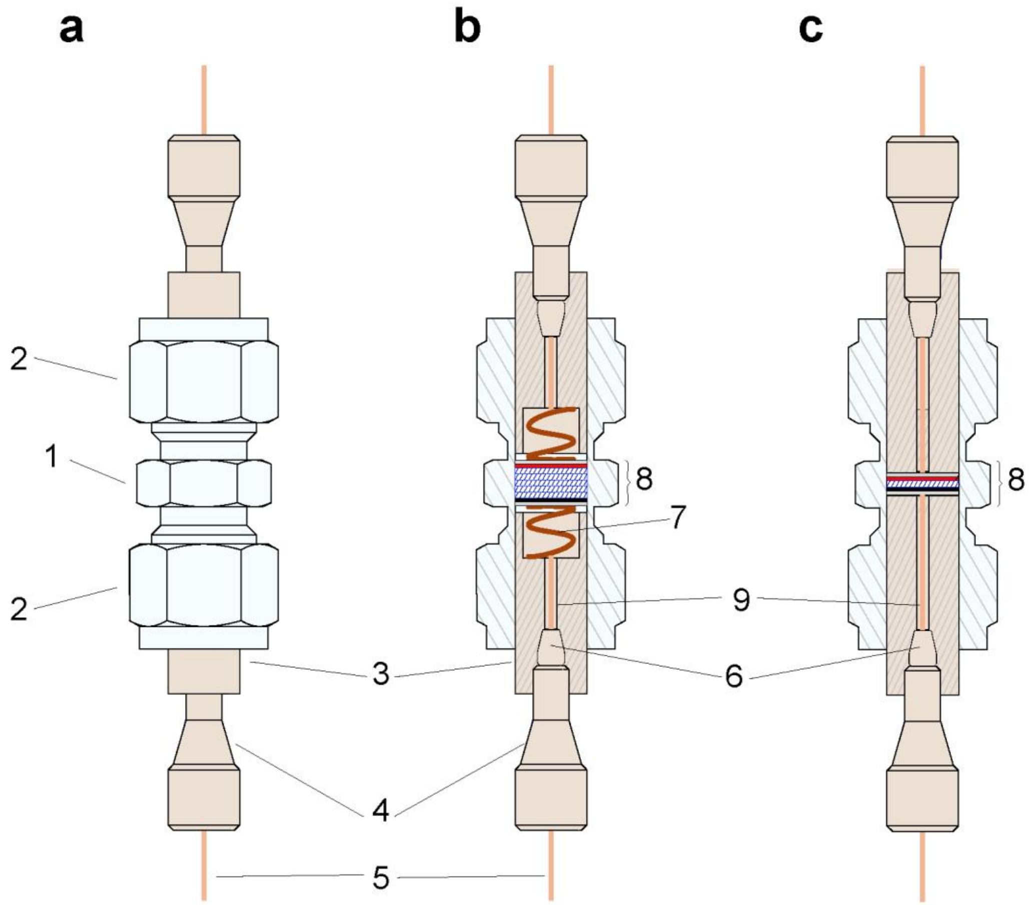

Supplementary Figure 1: Swagelok cell design. Exterior view (a) of the Swagelok cell and interior/cutaway views of the (b) sodium metal cell and (c) full-cell, with components labelled as follows: 1. 3/8" PTFE Swagelok union. 2. Swagelok nut. 3. custom-made PEEK plug (with recess only where spring was used (b)). 4. 1/16" PEEK HPLC nut. 5. Insulated copper wire. 6. 1/16" PEEK HPLC ferrule (VICI®). 7. Brass springs. 8. Electrode and separator stack. 9. Copper wire with PTFE sheath Sodium metal cell electrodes (b) were hard carbon (black) and sodium metal (red), separated by two or five glass microfibre separators. Full-cell electrodes (c) were hard carbon anode (black) and NaNFMSO cathode (red), separated one glass microfibre separator.

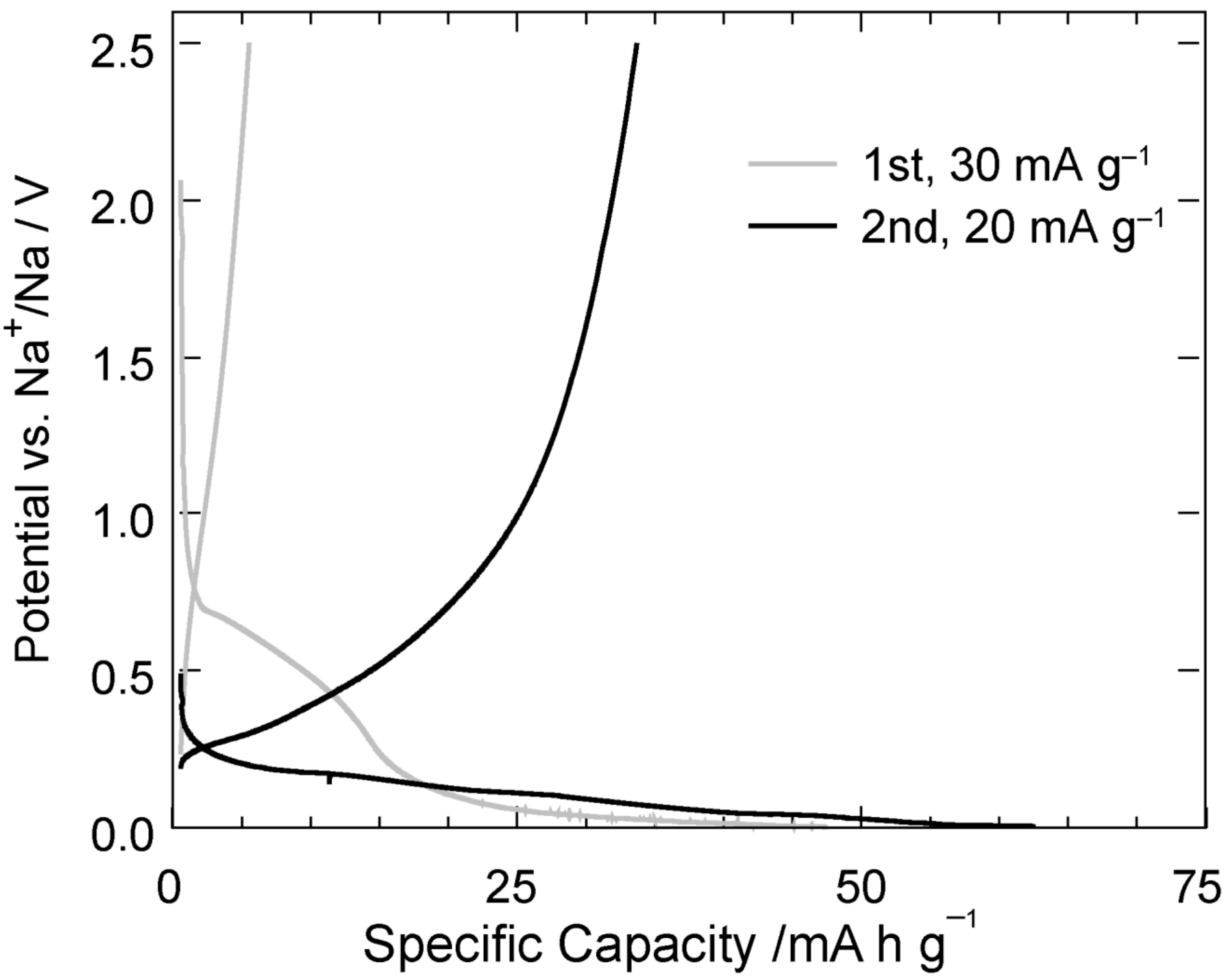

Supplementary Figure 2: Electrochemical Plots. Discharge/charge cycle (1st) at a specific current of 30 mA g^{-1}, performed outside the magnet prior to imaging, and a second cycle at 20 mA g^{-1}, performed inside the magnet during imaging experiments.

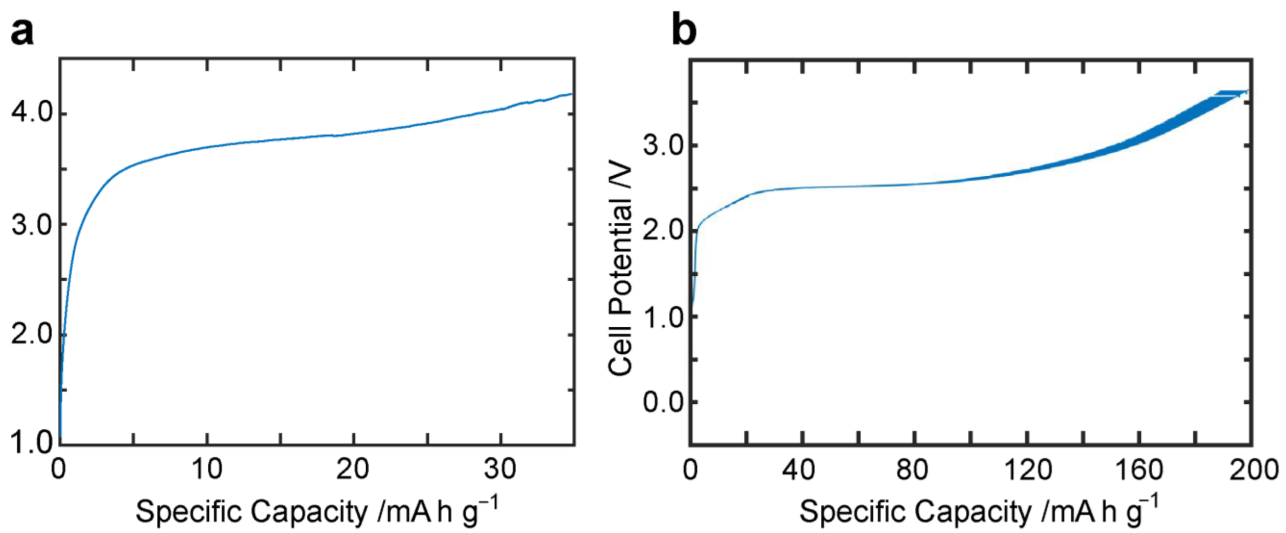

Supplemnetary Fig. 3: Full-cell Electrochemical Plots. Charge cycle (3rd) for the full cell with ^{23}Na NMR performed *in situ* (Fig. 7) at 86 mA g^{-1} (a). Formation cycle at 30 mA g^{-1} for the full cell with ^{23}Na NMR performed *in operando* (Fig. 5 and 6).

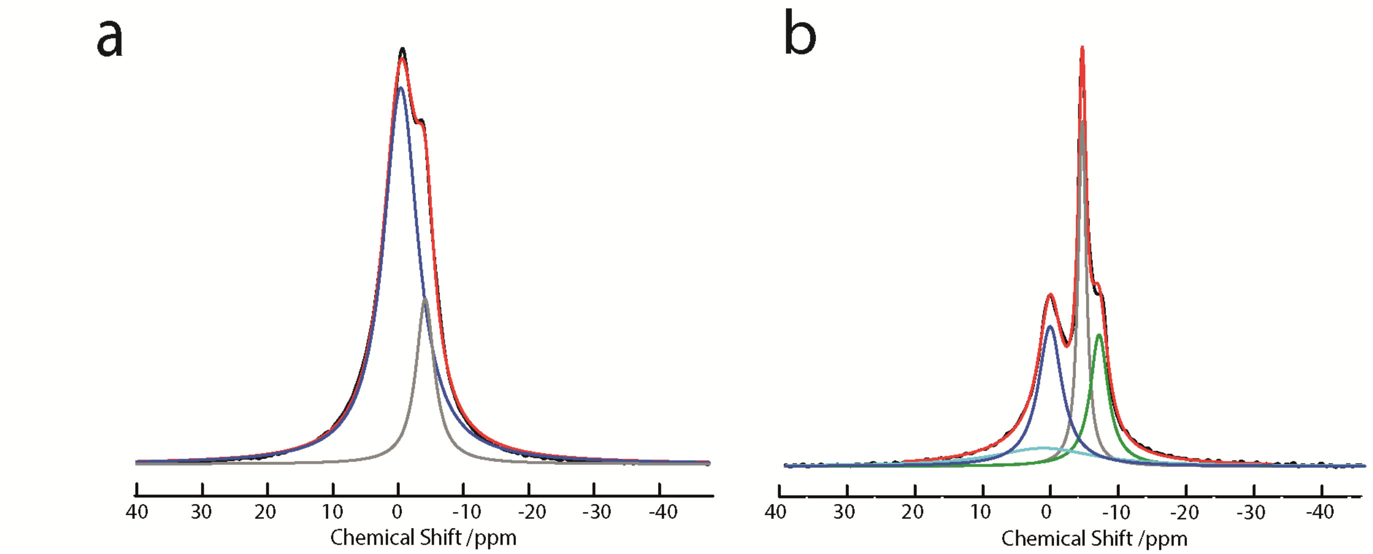

Supplementary Fig. 4: Deconvolution of ^{23}Na spectra for a pristine and post charge cycled battery. Deconvolutions for the ^{23}Na NMR spectra for solvated sodium in a pristine cell (a) and cell after a single discharge/charge cycle (b). The experimental spectrum is shown in black and the fit to the spectrum is given in the red. The individual peaks are shown for sodium in the electrolyte within the separators (dark blue), sodium in the electrolyte outside the cell (grey), sodium at the interface of the working electrode (green), and sodium with reduced mobility (pale blue), such as may be found in the solid-electrolyte interphase. The peak intensity for the sodium in the separator is larger in the sample in (a) because there are five separators in this cell and two in the cell for (b).

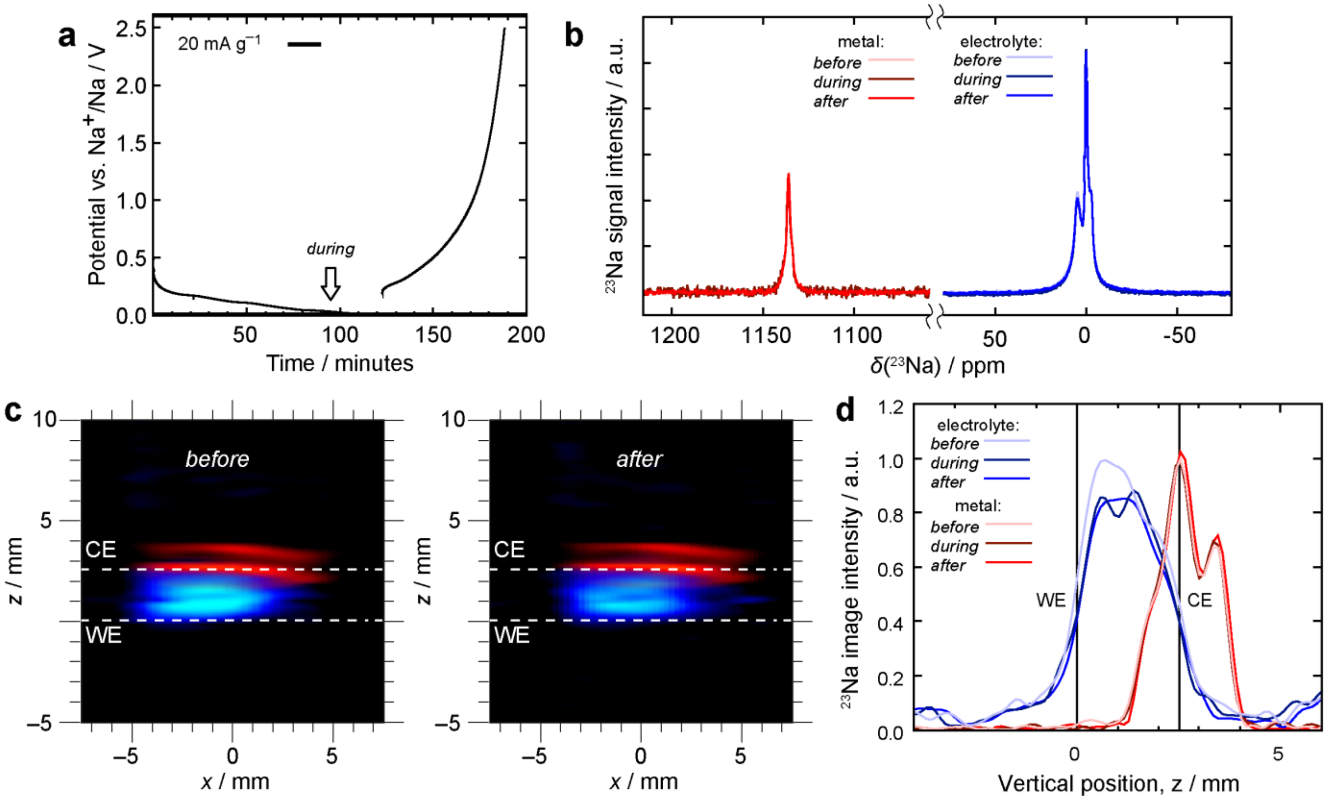

Supplementary Fig. 5: Electrochemistry, ^{23}Na Spectroscopy and Imaging during Charge Cycling. Chronopotentiometry plot (a) acquired during 2nd charge cycling at a specific current of 20 mA g^{-1}, with ^{23}Na spectra (b), 2D images (c) and 1D profiles (d) before, during and after cycling.

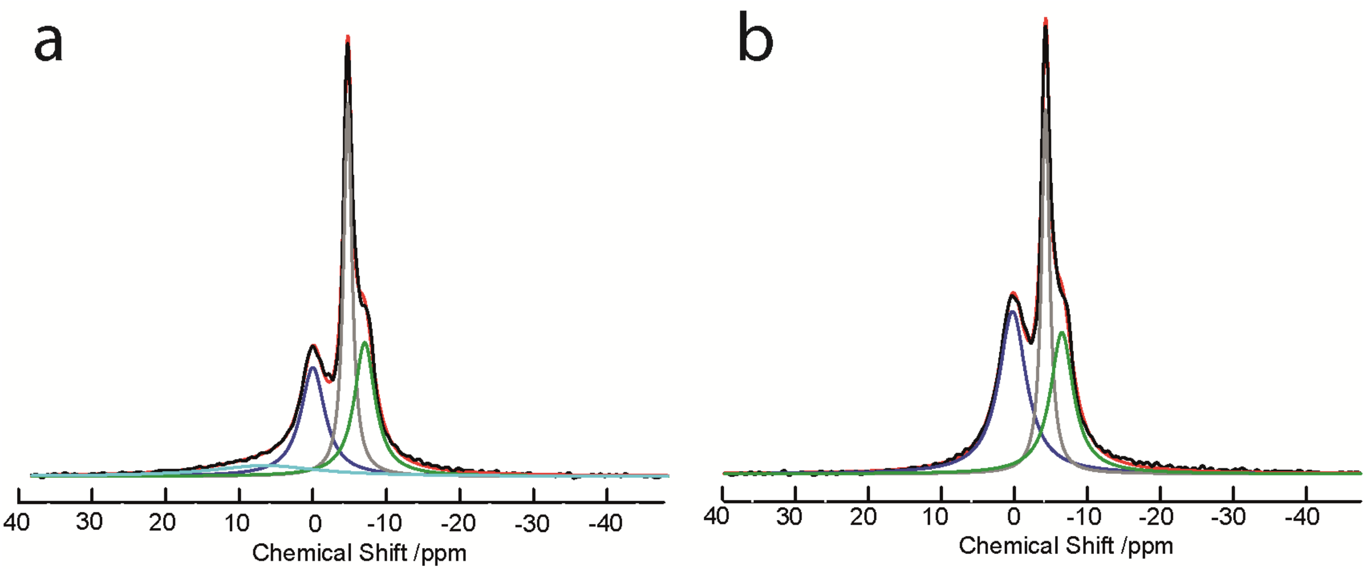

Supplementary Fig. 6: Deconvolution of ^{23}Na spectra for solvated sodium at the start and after continued plating. Deconvolutions for the ^{23}Na NMR spectra for solvated sodium in a cell (a) at the start of and (b) after continued plating (Fig. 3). The experimental spectra are shown in black and the fit to the spectrum is given in the red. The individual peaks are shown for sodium in the electrolyte within the separators (dark blue), sodium in the electrolyte outside the cell (grey), sodium at the interface of the working electrode (green), and sodium with reduced mobility (pale blue), such as may be found in the solid-electrolyte interphase.

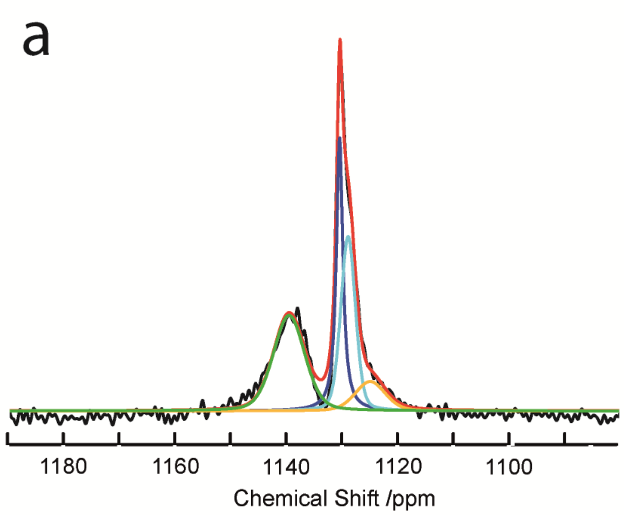

Supplementary Fig. 7: Deconvolution of ^{23}Na spectra for metallic sodium at the start and after continued plating. Deconvolutions for the ^{23}Na NMR spectra for metallic sodium in a cell at the start of (a) and continued plating (b) (figure 4). The experimental spectra are shown in black and the fit to the spectrum is given in the red. The individual peaks are shown in dark blue for the sodium counter electrode, light/dark green for sodium microstructures plated at approximately 90˚ to the B_0 field, and light blue and orange for sodium interfaces plated (on WE) or pitted (on CE), with an approximately 0˚ orientation with respect to the B_0 field.

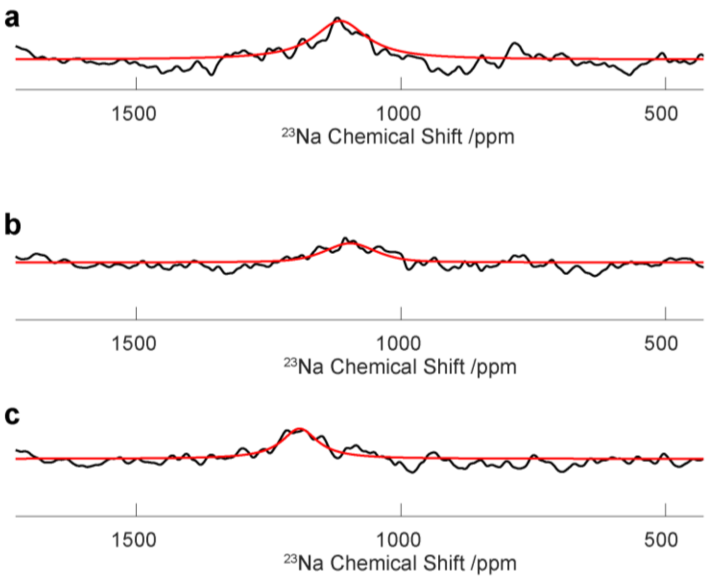

Supplementary Fig. 8: Metallic region of ^{23}Na full spectra acquired *in operando*. After charging at 30 mA g^{-1} for 2 h (a), 2.5 h (b), and 3 h (c).

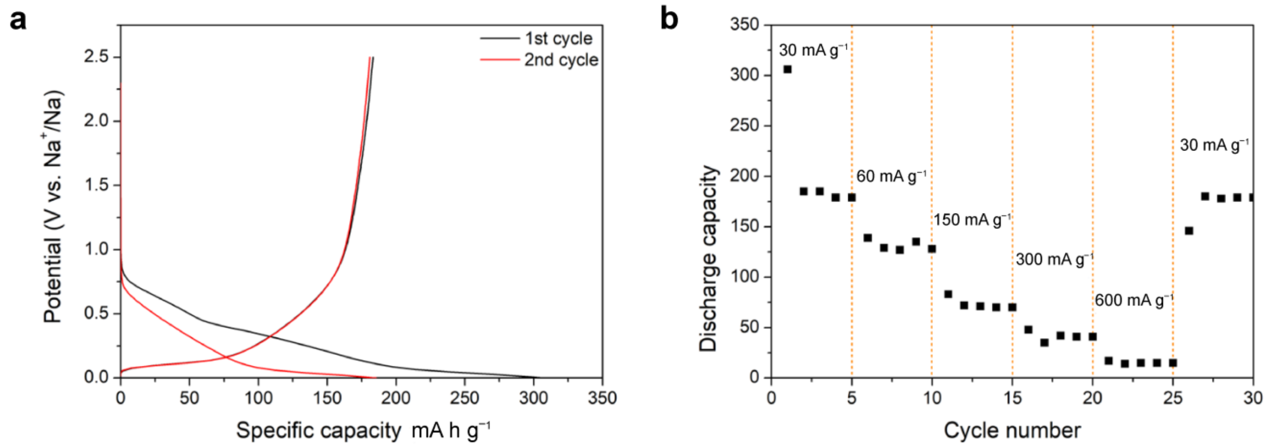

Supplementary Fig. 9: Electrochemical performance of hard carbon sodium metal cell. (a) Potential profiles of the first two sodiation/desodiation cycles of hard carbon at 30 mA g^{-1}; (b) Rate capability of hard carbon at different current densities.

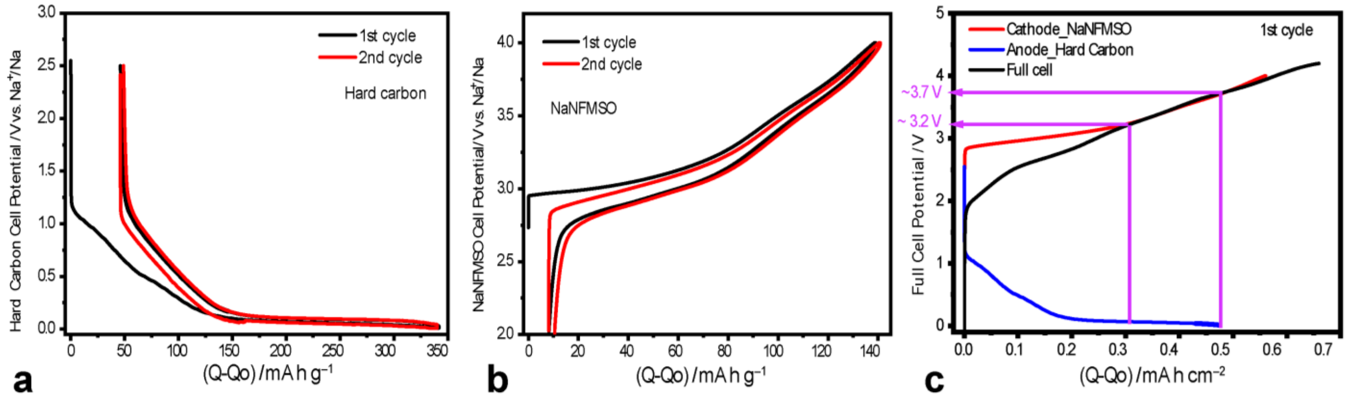

Supplementary Fig. 10: Sodium metal cell electrochemical plots for full-cell development. Charge-discharge curves of cathode (a) and anode (b) at a specific current of 20 mA g^{-1}. Black and red curves represent the 1st and 2nd cycle. Full cell cycled at a specific current of 100 mA g^{-1} and sodium metal cell electrochemical plots(c). Red, blue and black curves represent the charge profile of cathode, anode and the full-cell. Dashed lines illustrate regions where different insertion mechanisms are expected to take place.

Supplementary Tables

Supplementary Table 1: Measured ^{23}Na NMR parameters of battery components.

Parameter:	Metallic Na	Dissolved Na^+ in separator
Frequency, *f*	105.9761111 MHz	105.8561019 MHz
T_1	19 ± 2 ms	2 ± 1 ms
T_2	7.5 ± 0.5 ms	3 ± 1 ms
T_2*	1 ± 0.5 ms	1 ± 0.5 ms

Supplementary Table 2: Image geometry for the 2D spin-echo and gradient-echo sequences.

	**Spin-echo imaging (metallic Na)**	**Gradient-echo imaging (dissolved Na^+)**
**Vertical direction (*z*):**		
FOV / mm	59.0	59.0
Frequency-encode points	256	256
Resolution / µm	231	231
**Horizontal direction (*x*):**		
FOV / mm	16.9	19.5
Phase-encode steps	16	16
Resolution / µm	1050	1220

Supplementary Table 3: Deconvolution values for electrolytic sodium in ^{23}Na NMR spectra in sodium metal cell

Peak	After 1st charge cycle			After 2nd charge cycle			After discharge at a rate 150 mA g^{-1}			After discharge at a rate 600 mA g^{-1}		
	δ /ppm	Δν /Hz	%	δ /ppm	Δν /Hz	%	δ /ppm	Δν /Hz	%	δ /ppm	Δν /Hz	%
1	–7.0	334	23	–7.0	349	26	–7.0	358	28	–7.0	399	30
2	–5.0	152	27	–5.0	149	30	–5.0	146	32	–5.0	144	28
3	0.0	448	32	0.0	397	24	0.0	421	27	0.0	480	42
4	1.0	1901	18	1.0	2399	20	6.5	1878	12			

Supplementary Table 4: Deconvolution values for metallic sodium in ^{23}Na NMR spectra in sodium metal cell

Peak	After 1st charge cycle			After 2nd charge cycle			After discharge at a rate 150 mA g^{-1}				After discharge at a rate 600 mA g^{-1}			
	δ /ppm	Δν /Hz	%	δ /ppm	Δν /Hz	%	δ /ppm	Δν /Hz	%	Gaussian %	δ /ppm	Δν /Hz	%	Gaussian %
1	1131	309	100	1131	293	87	1125	703	10	75	1126	1259	9	75
2				1141	1413	13	1129	342	29	0	1129	354	7	75
3							1131	153	27	75	1131	127	4	0
4							1141	736	34	75	1136	585	33	75
5											1140	1091	48	75

Supplementary Table 5: **Deconvolution values for *in situ* ^{23}Na NMR spectra in full-cell after charging**

Peak	After Charge				After 4 Hours				After Discharge		
	δ /ppm	Δν /Hz	%	Gaussian %	δ /ppm	Δν /Hz	%	Gaussian %	δ /ppm	Δν /Hz	%
1	−157	9901	4	50							
2	−49	9529	13	50	−10	3398	7	0			
3	9	3012	67	0	9	2696	64	0	11	3030	100
4	486	16066	16	100	652	22976	29	100			

Supplementary Table 6: Deconvolution values for selected *in operando* ^{23}Na NMR spectra in full-cell duration formation charge cycle

Peak	Pristine Cell			After Charging for 1.5 Hours				After Charging for 4.5 Hours			
	δ /ppm	Δv /Hz	%	δ /ppm	Δv /Hz	%	Gaussian %	δ /ppm	Δv /Hz	%	Gaussian %
1	−7	633	9	−198	6264	3	50	−148	13367	15	0
2	0	293	49	−64	11023	18	0	−43	6205	12	0
3	8	1052	42	0	3239	52	0	0	2889	53	0
4				133	109	8	50	339	18298	20	100
5				1109	12632	20	0				

Supplementary Methods

Swagelok Design: The cell design for the SIB was a modification of a "Swagelok cell" design commonly used for battery testing, where stainless steel parts have been substituted with non-magnetic ones. The bore of the Swagelok union was drilled to an inner diameter of 3/8". Supplmentary Fig. 1 shows a schematic diagram of the cell. For the sodium metal cell (b), the electrode/electrolyte stack (described fully in the main text) was held between two phosphor bronze springs (Small-Order Springs & Pressings, Ltd., UK), which are recessed into custom-made, polyether ether ketone (PEEK) inserts. For the full cell, the brass springs were omitted and the PEEK plug replaced. Channels within these inserts allow electrical leads (copper wire) to pass through, and an airtight seal around the wire is achieved using a PEEK nut/ferrule assembly commonly used for HPLC assemblies (i.d. 1/16'' = 1.6 mm, VICI®) in combination with a PTFE sheath fitted tightly around the wire. The compressibility of the PTFE sheath creates a tighter, more stable seal than simply the PEEK on the bare copper. The cell is held together using a PTFE union tube fitting (i.d. 3/8'' = 9.5 mm, Swagelok®), which creates an airtight seal around the inserts using a ferrule/back-ferrule assembly.

Magnetic Resonance Experiments: ^{23}Na nuclear magnetic resonance spectra (NMR) and images (MRI) of the SIB were acquired on a Bruker 9.4 T magnet using a 25 mm WB40 ^{23}Na radiofrequency probe for the sodium metal cell measurements and 25 mm WB40 $^{1}H/^{23}Na$ dual resonance radiofrequency probe for the full-cell measurements. Initial positioning of the sample and correction of magnetic field inhomogeneities (shimming) for the sodium metal cell measurements were performed using a 25 mm WB40 ^{1}H probe for detection of the electrolyte solvent (EC:DMC) rather than the ^{23}Na. Following this setup, the sodium metal cell was transferred to the ^{23}Na probe using a sample holder that enabled fixed and repeatable positioning. Optimisation of the full-cell was performed using the ^{1}H of the ethylene carbonate:diethyl carbonate (EC:DEC) electrolyte, in the same probe as ^{23}Na measurements.

The NMR frequency of the dissolved Na^+ in the electrolyte of the pristine cell was assigned to 0 ppm, and relative to this internal reference, the measured Knight shift of the bulk Na metal electrode was 1131 ppm. The optimal tuning and matching of the RF probe, as well as calibration of 90° pulses, were determined at each frequency separately. The NMR relaxation times of the metal/electrolyte signals in the pristine cell were measured using inversion recovery (T_1), CPMG (T_2), and FID (T_2*) acquisitions and are given in Supplementary Table 1.

NMR spectra were acquired in separate measurements around the 0 ppm and 1131 ppm frequencies. The acquisition was performed with a 10 µs dwell time and 1024 points, using 2048 averages and a repetition time of T_R = 0.1 s for a scan duration of 3 min 24 s. Fourier transformation was done without filtering. Two-dimensional (2D) ^{23}Na MR images were acquired using a spin-echo based sequence. Due to the short relaxation times, no slice selection was used, and images are therefore projections of the entire cell thickness onto a vertical plane. All images were acquired with frequency encoding in the vertical direction (z, along the axis of the cell) using a 5 µs dwell time and 256 points, with an asymmetric echo acquisition scheme to optimise image signal intensity. Phase encoding was done in the horizontal direction (x) using 16 steps, and the optimised phase encoding duration for each image type resulted in different field-of-view (FOV) for each one (summarised in Supplementary Table 2). The experimental parameters for the 1D profiles are the same, but with no phase encoding. The 2D scans were acquired with a repetition time of T_R = 0.1 s and 1024 averages, requiring 28 minutes for each. One-dimensional (1D) profiles at a zero phase encoding gradient were also employed for faster, *in operando* imaging, and were acquired using 2048 signal averages, resulting in a scan duration of only 3 min 35 s.

Image processing was performed using Prospa software (Magritek Ltd.). The 2D data matrices were zero-filled to 512 × 64 points and the 1D matrices filled to 512 points; conjugate symmetry was exploited to reconstruct data missing due to the asymmetric frequency-encoding; and a sine-bell squared filter was applied to minimise any Gibbs ringing artifacts in the images. “Negative images” of sodium microstructures were produced from the ^{1}H 3D MRI data by only displaying voxels below a threshold intensity, which identified regions where the electrolyte was absent and hence enabled the detection of metallic sodium microstructures within the electrolyte. The threshold value was chosen just above the noise level, at a level of 7.4% of the maximum image intensity.
NMR spectra covering the full spectral range (0 – 1200 ppm), including signal from dissolved (0 ppm) to metallic sodium signal (ca. 1200 ppm) were acquired using a spin echo sequence using pulse lengths of 2 μs and 4 μs, collecting 512 points and 32768 signal averages, with a repetition time of 0.05 s resulting in experiment times of 27 mins 18 s.

Electrochemistry: Electrochemical measurements were performed using an Ivium Octostat 5000 potentiostat connected to the cell in a 2-electrode configuration.

Sodium metal cell experiments: The open circuit voltage was recorded and an initial discharge/charge cycle was performed at a specific current of 30 mA g^{-1} outside the magnet prior to imaging. This is shown in Supplmentary Fig. 2 along with a second cycle at a specific current of 20 mA g^{-1}, which was performed inside the magnet.

From the electrochemical data of the sodium metal cell, the following charge is transferred: Cycle 1 discharge: +47 mA h g^{-1}, Cycle 1 charge: –5 mA h g^{-1}, Cycle 2 discharge: +41 mA h g^{-1}, Cycle 2 charge: –22 mA h g^{-1}; indicating not all of the sodium is removed in the first charge cycle (30 mA g^{-1}) and that during the second, slower, charge cycle (20 mA g^{-1}) more sodium is extracted from the carbon electrode than in the first. We believe the difference in electrochemical behavior of the carbon electrode material, between this study and previous measurements in coin cells, is most likely due to the different cell configuration used in this study.

Full-cell experiments: To improve the electrochemical behaviour observed, and detect the presence of metallic and quasimetallic sodium, the Swagelok cell was optimised (Supplementary Fig. 9) and a full-cell configuration was adopted. The sodium metal electrode was replaced with a NaNFMSO cathode and the hard carbon electrode was replaced with a hard carbon electrode. Additionally, the number of separators was reduced to one and the springs were removed, to ensure even compression across electrodes and separators.

The open circuit voltage was recorded, and a formation cycle was performed, *in operando*, in the magnet at a specific current of 30 mA g^{-1} while ^{23}Na NMR spectra were collected. The electrochemical behavior is shown in Supplementary Fig. 3. For the *in situ* observation of the quasimetallic species, the cell was cycled twice between 1–4.2 V before charging to a potential of 4.18 V, at a specific current of 86 mA g^{-1}, and was not held at this potential during the acquisitions. Any sodium plating that may have occurred at this potential was below the level of detection.

The high specific current (86 mA g^{-1}) used in Supplementary Fig. 3(a) shows the high polarisation in the cell configuration (higher voltage and low specific capacity), therefore much lower specific current (30 mA g^{-1}) was required to achieve the full capacities of the hard carbon Supplementary Fig. 3(b).

In the full cell work, we used a commercial hard carbon, which typically has a particle size of 5–7 μm. Previous work regarding these materials has been published[2-4]. To avoid Na metal plating, in the full cell, the negative and positive electrode capacity is balanced, using Supplementary Eq. 1, such that there is enough capacity in the negative electrode to accept the sodium ions from the positive electrode. This balancing calculation[5] is based on the assumption that the ratio Q_{dis} is greater than or equal for negative and positive electrodes ($(N:P)_Q$ capacity ratio ≥1:1) in mA h for each electrode.

$$Q_{dis} = q \cdot m \qquad (1)$$

where q (in mA h g^{-1}) is the reversible specific capacity and m (in g) is the active mass in the electrode.

In this cell system, we wanted to study sodiation in the hard carbon, therefore we have created a cell with an excess of sodium in the positive electrode. Therefore, to be able to detect the presence of metallic and quasimetallic sodium, the full cell was optimised by adjusting the mass loading of the negative and positive electrodes to 1:3 giving a capacity balance of N:P as 1:1.3.

As presented in Supplementary Fig. 10a and b, the positive and negative sodium metal cells were cycled between 2.0–4.0 and 0.01–2.5 V *vs* Na/Na^+ and delivered a reversible specific capacity of 130 mA h g^{-1} and 295 mA h g^{-1} respectively. In Supplementary Fig. 10c, the negative and positive voltage charge profiles are mapped onto the full cell voltage profile. This shows that full sodiation of the hard carbon is completed at 3.7 V. If a higher voltage is applied, it will result in sodium plating on the hard carbon. Voltage control of the full cell enabled us to investigate the specifics of the sodiation processes in this hard carbon, and removed the resistances observed when using a sodium metal counter electrode.

^{23}Na NMR Spectroscopy: Spectral fitting was performed using DM Fit software[1] using a combination of Lorentzian and Gaussian functions as indicated in Supplementary Tables 3–6. A minimum number of peaks were selected, to enable the spectrum to be adequately fitted.

The images shown in Supplementary Fig. 5, taken before, during and after charge cycling, do not show major changes in the distribution of Na species within the cell. However, a slight reduction in the ^{23}Na signal intensity on the right-hand side of the electrolyte suggests that there is possibly the formation of degradation products in this region.

Materials: Electrochemical studies of the amorphous carbon working electrode from the sodium metal cell were performed in a coin cell and found to exhibit typical sloping and plateau regions within the potential vs. capacity profiles which are characteristic of hard carbons and have an overall reversible capacity of 190 mA h g^{-1} (Supplementary Fig. 9a). At successively higher current densities, the capacity is found to drop to 50 mA h g^{-1} (when cycled at 1C; Supplementary Fig. 9b) but recovers the original values when cycled once again at 30 mA h g^{-1}. The decrease in capacity at high current densities is due to an increase in various internal resistances within the cell; furthermore, at very high current densities and close to 0 V, sodium plating is believed to occur preferentially to intercalation at the working electrode, which will have an adverse effect on battery performance.

Supplementary References

1. Massiot, D. et al., *Magn. Reso. Chem.* **40**, 70–76 (2002).
2. Robinson, J. B. *et al.* Microstructural Analysis of the Effects of Thermal Runaway on Li-Ion and Na-Ion Battery Electrodes. *J. Electrochem. Energy* **15**, 0110101–0110109 (2018).
3. Ledwoch, D., Brett, D. J. L. & Kendrick, E. The Performance of Hard Carbon in a Sodium Ion Battery and Influence of the Sodium Metal in Observed Properties. *ECS Trans.* **72**, 17–22 (2016).
4. Robinson, J. B. *et al.* Multiscale tomographic analysis of the thermal failure of Na-Ion batteries. *J. Power Sources* **400**, 360–368 (2018).
5. Kasnatscheew, J. *et al.* A Tutorial into Practical Capacity and Mass Balancing of Lithium Ion Batteries. *J. Electrochem. Soc.* **164**, A2479–A2486 (2017).
